# Depot medroxyprogesterone acetate and breast cancer: a systematic review

**DOI:** 10.1007/s00404-023-07265-5

**Published:** 2023-11-15

**Authors:** Aline Zürcher, Laura Knabben, Marc von Gernler, Petra Stute

**Affiliations:** 1 University of Bern, Bern, Switzerland; 2 Department of Obstetrics and Gynecology, Inselspital, University Clinic Bern, University of Bern, Friedbühlstrasse 19, 3010 Bern, Switzerland; 3 Medical Library, University Library of Bern, University of Bern, Bern, Switzerland

**Keywords:** Breast cancer, Progestin-only contraceptives, Injectable contraceptives, Depot medroxyprogesterone acetate, Depo-Provera

## Abstract

**Purpose:**

Short-acting progestin-only injectables containing depot medroxyprogesterone acetate (DMPA) are a safe method of contraception. Although DMPA has been available for several decades, there is little data on its influence on the risk of breast cancer. Hence, the aim of this paper was to provide an overview of the existing studies and create clarity regarding a possible association with breast cancer.

**Methods:**

Literature searches were executed in MEDLINE, Embase, the Cochrane Library, ClinicalTrials.gov and ICTRP. Search terms were related to DMPA and breast cancer. After elimination of duplicates, 3′850 studies were identified and assessed according to inclusion and exclusion criteria. Finally, ten studies were selected and included in this review.

**Results:**

All the selected papers were case–control-studies, except for one pooled analysis and one study comparing observed and expected number of cancer cases.

Most of the included studies found no overall elevated breast cancer incidence in DMPA users, only one study found a slightly increased risk and two studies concluded with a significant increase for the overall breast cancer risk.

**Conclusion:**

There is little evidence that DMPA may increase the overall risk for breast cancer. However, the incidence of breast cancer is possibly increased in current and more recent users, especially in women younger than 35 years. Long-term use did not result in any risk increase. Nevertheless, further studies will be necessary to confirm these findings and weigh up the individual risks and benefits of this contraceptive method.

## What does this study add to the clinical work

**Table:** 

Findings of this systematic review will greatly add to our current knowledge on DMPA and its influence on the risk of breast cancer. As it is a widely used contraceptive method, extensive knowledge about its (side) effects is essential. This makes it easier to weigh up the individual risks and benefits in clinical use.	

## Introduction

In 2019, 3.9% of women aged 15–49 years were using injectables—in concrete figures: 74 million people worldwide were relying on this short-acting contraceptive method. Injectables containing depot medroxyprogesterone acetate (DMPA) are used mainly in sub-Saharan Africa and South-Eastern Asia. The prevalence is over 20% in Indonesia, Madagascar, Malawi, Namibia and South Africa. Cultural factors, practicability and availability, but also costs probably play a role in the choice of contraceptive method. Meanwhile, injectable contraception is less common in Europe, where the prevalence is only 0.5%. Instead, the pill and the male condom are the most commonly used contraceptive methods there [1]. Reasons for less use of DMPA in Europe could include easier availability of other, more accepted contraceptive methods and different medical practices or professional recommendations. In addition, Europeans also seem to have more concerns about side effects.

Within the progestin-only injectables containing DMPA, Depo-Provera is most used. It is injected every 3 months and a very safe contraceptive method, as the contraceptive level is maintained for at least 14 weeks. DMPA relies on higher peaks of progestin which inhibits ovulation, thickens the cervical mucus and alters the endometrium. As only the luteinizing hormone (LH) surge is suppressed, follicular growth is still maintained by the follicle-stimulating hormone (FSH) and estrogen levels are comparable to the early follicular phase of a normal menstrual cycle [2].

DMPA is a very efficient contraceptive method, and the administration involves little effort. However, use of DMPA is often discontinued due to side effects such as irregular menstrual bleeding, weight gain, mood changes and increased headaches [3]. Additionally, DMPA injections showed a detrimental effect on bone density [4].

Breast cancer is the leading cause of global cancer incidence, with 2.3 million new cases in 2020. This represents 11.7% of all cancer cases worldwide. Several risk factors have been identified, including hormonal contraceptives [5].

Even though Depo-Provera has been available since the 1960s, there is little data on its influence on breast cancer risk. As the results on the association of other progestin-only methods with breast cancer were mixed, there was still concern that DMPA might increase the breast cancer incidence in women. The aim of this paper was therefore to provide an overview of the existing studies concerning this topic and create clarity regarding a possible link between DMPA and breast cancer.

## Methods

To identify all potentially relevant documents on the topic, complex literature searches were designed and executed for the following information sources: MEDLINE, Embase, Cochrane Library, ClinicalTrials.gov and ICTRP.

An initial search strategy was developed in MEDLINE by a medical information specialist and tested against a list of core references to see if they were included in the search result. After refinement and consultation, complex search strategies were set up for each information source based on database-specific controlled vocabulary (thesaurus terms/subject headings) and textwords. Synonyms, acronyms, and similar terms were included in the textword search. No limits have been applied in any database considering study types, languages, publication years or any other formal criteria. All searches were run on July 25^th^, 2022.

The following search concepts were applied according to the PICO framework: 1. "Breast cancer" as the population and 2. "Depot medroxyprogesterone acetate" as an intervention. Index terms, synonyms, acronyms, similar terms and drug names were used for the search in MEDLINE, Embase and the Cochrane library. Studies concerning exclusively animals, plants or fungi were excluded from the searches in Medline and Embase by using a double-negative search strategy based on the "Humans only" filters by Ovid. The searches in the trial registers were performed using free text search terms and acronyms only. The detailed final search strategies are published in the digital library searchRxiv [6–10].

All identified records were imported into EndNote, exported as RIS and deduplicated using the online tool Deduklick (https://www.risklick.ch/products/deduklick/) [11].

The systematic review was registered in the international prospective register of systematic reviews (PROSPERO) database.

**Fig. 1:**
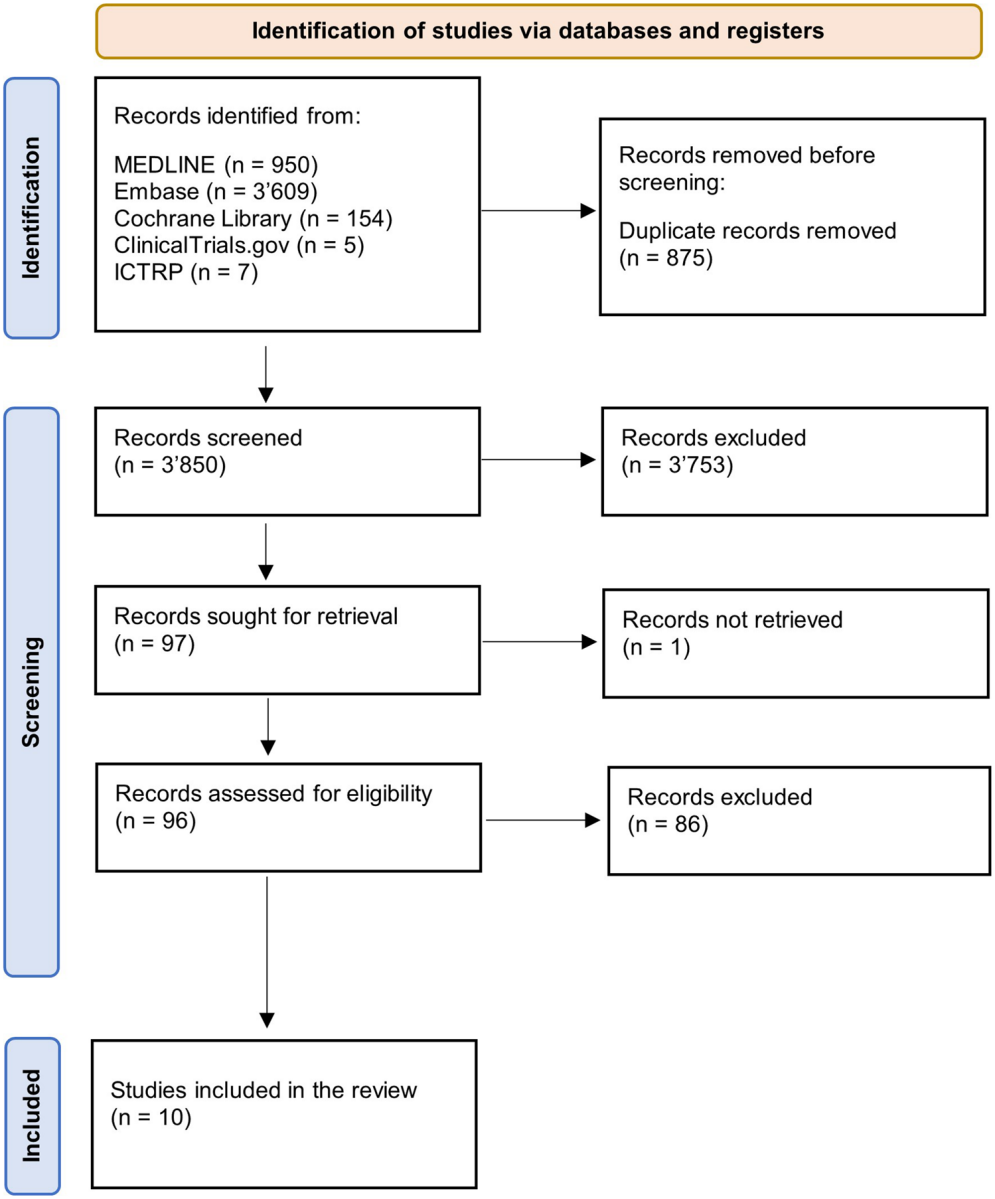
PRISMA flow chart of the screening process

Hereafter, 3′850 studies were included in the screening process and inclusion and exclusion criteria were applied, according to the Preferred Reporting Items for Systematic Reviews and Meta-Analyses (PRISMA) statement guidelines. Inclusion criteria regarding the study population were women using DMPA, exclusion criteria were women known to be at high risk for developing breast cancer or exposed to hormone replacement therapy. In two screening rounds, the studies were sorted out by two reviewers, so that in the end ten papers remained and were included in this review (Fig. 1).

## Results

In total, 3850 studies were found with the literature search above described. The studies were selected in two screening rounds according to the four-eye principle, so that in the end ten studies remained and were included in this review (Table 1).

**Table 1:** Included studies with more detailed results and summary

Author	Results	Summary
Greenspan, 1980 [13]	**RR = 1.0 (the authors did not calculate the 95% CI)**	Short-term use of DMPA was not associated with any increased risk of breast cancer
Lee, 1987 [14]	**RR = 2.6 (95% CI 1.4–4.7)**	Ever use of DMPA seemed to be associated with an elevated risk of breast cancer. The risk increase was also present in the group who was exposed to DMPA for less than a year. However, long-term use showed no elevated risk
Li, 2012 [15]	**OR = 1.2 (95% CI 0.9–1.6)** Women using DMPA for ≥ 12 months: OR = 2.2 (95% CI 1.2–4.2)	Neither ever use nor recent use of DMPA increased the breast cancer risk. Nonetheless, recent use of DMPA for ≥ 12 months showed an increased risk. The elevated risk seemed to dissipate after discontinuation
Liang, 1983 [21]	**RR = 0.7 (95% CI 0.3–1.4)**	DMPA use did not increase the breast cancer risk
Paul, 1989 [16]	**RR = 1.0 (95% CI 0.8–1.3)** Women aged 25–34 years: RR = 2.0 (95% CI 1.0–3.8) Women who had used DMPA for two years or longer before age 25: RR = 4.6 (95% CI 1.4–15.1)	The study found no overall increased breast cancer risk. However, the risk was increased in women who had used DMPA before the age of 35 and in those who used it for at least two years before age 25 or who had used it recently
Shapiro, 2000 [17]	**RR = 0.9 (95% CI:0.7–1.2)** Currently exposed women: RR = 1.6 (95% CI 1.1–2.3)	DMPA did not elevate the overall risk of breast cancer. Prolonged duration of use did not seem to increase the risk either Current use of DMPA raised the breast cancer incidence but was inconsistent across the different age groups
Skegg, 1995 [20]	**RR = 1.1 (95% CI 0.97–1.4)** Women who had initiated DMPA use within the last five years: RR = 2.0 (95% CI 1.5–2.8) Current DMPA users younger than 35 years: RR = 2.1 (95% CI 1.1–3.8)	No overall increased risk of breast cancer was found in DMPA users However, women who started using DMPA within the previous five years appeared to have an increased risk of breast cancer
Strom, 2004 [18]	**OR = 0.9 (95% CI 0.7–1.2)** Current users: OR = 0.7 (95% CI 0.4–1.3)	The study showed no overall increased breast cancer risk, as well as among current users of injectable contraceptives and women who began at a young age
Urban, 2012 [19]	**OR = 1.31 (95%CI 1.03–1.65)** Current users: OR = 1.83 (95% CI 1.31–2.55)	The use of injectable contraceptives was associated with a transiently increased risk of breast cancer among current and more recent users
WHO Collaborative Study of Neoplasia and Steroid Contraceptives, 1991 [12]	**RR = 1.21 (95% CI 0.96–1.52)**	The use of DMPA did only slightly increase the overall risk for breast cancer. The breast cancer risk did not increase with duration of use but was higher in women who had been exposed to the drug initially within the previous 4 years. These women tended to be younger than 35 years

The selected studies were all case–control studies [12–19], except for one pooled analysis [20] and one study which compared the observed and expected number of cancer cases [21].

The research was performed in Thailand, Mexico and Kenya [12], the United States [13, 15, 18, 21], Costa Rica [14], New Zealand [16] and South Africa [17, 19]. The pooled analysis included data from Thailand, Mexico and Kenya, as well from New Zealand [20].

Primary endpoints in all papers were the determination of a possible influence of DMPA on the breast cancer risk. Some studies also examined oral contraceptives (OC) [14, 17, 19] and progestin-only implants [18] or additionally focused on other types of cancer [19, 21].

The size of study cohorts ranged from only 30 women [13] to 4575 cases [18]. Duration of use was between one single injection and more than 10 years (> 40 injections in total) [17].

Seen as a whole, most of the included women were premenopausal and in the reproductive age, with outliers below (11 years [21]) and above (79 years [19]).

**Fig. 2:**
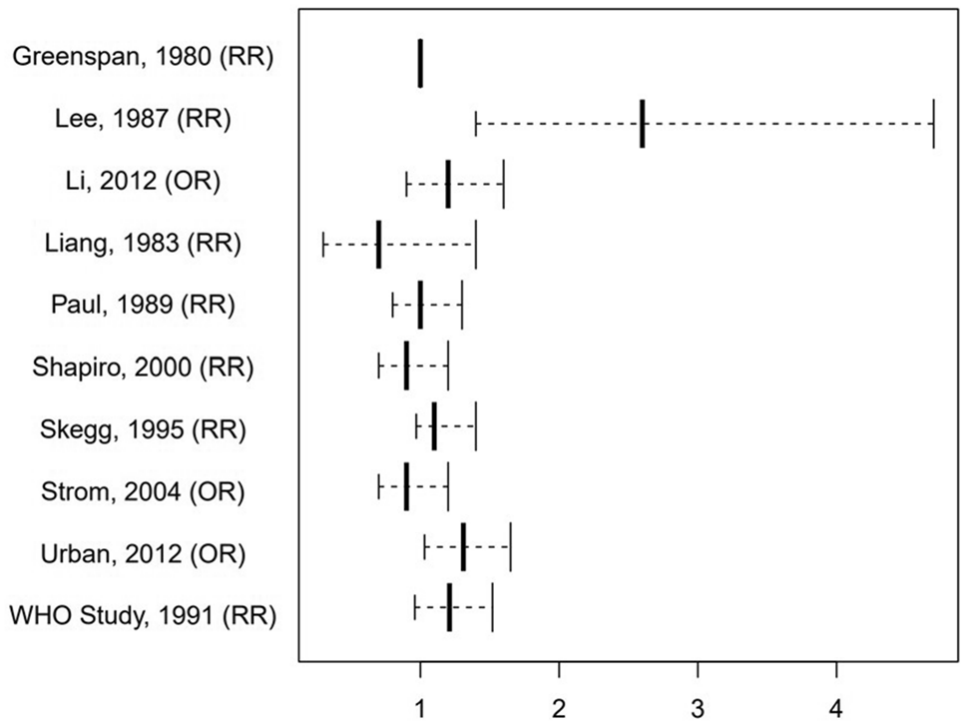
Graphical representation of the overall RR/OR of ever use of DMPA

Seven studies concluded with no increased overall breast cancer risk for DMPA users [13, 15–18, 20, 21]. However, many found a higher breast cancer incidence for certain groups of current or more recent users [12, 15–17, 19, 20]. Three studies reported a slightly or even significantly elevated overall risk of breast cancer among DMPA users [12, 14, 19] (Fig. 2).

Women receiving DMPA tended to be younger and more likely to be premenopausal than non-users, and they were more likely to have been previously exposed to OCs. Additionally, users of injectable contraceptives were more often black and from lower income groups.

### Overall breast cancer risk

Seven studies found no overall increased incidence of breast cancer in DMPA users, with relative risks (RR) ranging from 0.7 [21] to 1.2 [15].

One study found a slightly statistically significant increased RR of 1.21 (95% CI 0.96–1.52) [12]. Two studies concluded with a more clearly overall elevated risk and found an odds ratio (OR) of 1.31 (95% CI 1.03–1.65) [19] and a RR of 2.6 (95% CI 1.4–4.7) [14] respectively. However, the latter only studied a small cohort, with only 19 of the 171 breast cancer cases having ever used DMPA. Moreover, the same study found no increased incidence of breast cancer for long-term users.

### Breast cancer risk in current and recent DMPA users

While the overall breast cancer risk did not seem to be elevated for the most part, several studies concluded that the risk tended to be increased in current and recent users [12, 15–17, 19, 20]. Some authors specified that the elevated risk for current users was only evident for women who received DMPA for more than 12 months [15]. The risk increase for women currently using DMPA was inconsistent across the different age groups and was especially evident when they were younger than 35 years, with RRs up to 2.1 (95% CI 1.1–3.8) [20]. However, the elevated risk tended to decline again after cessation, regardless of the duration of use.

Only one study explicitly found no risk in current users, OR = 0.7 (95% CI 0.4–1.3). Current use was defined as women who were exposed to injectable contraceptives within one year of the reference date. It should be noted that although the study was quite large, the number of cases was rather small, with only 58 women ever receiving DMPA [18].

### Long-term use of DMPA

In general, longer duration of DMPA use did not seem to increase the risk of breast cancer. However, one study put this result into perspective: Although no overall increase with duration of use was found here either, women who had received DMPA for 6 years or longer had a higher risk, RR = 3.7 (95% CI 0.63–21.5). Additionally, there were indications that this risk was even higher in women who first used DMPA before the age of 25 years or before the first full term pregnancy. Again, the numbers of studied women who had used DMPA long-term was, however, small [16].

## Discussion

To sum up, seven studies found no overall increased incidence of breast cancer in DMPA users. With a RR of 1.21 (95% CI 0.96–1.52), the WHO Study found a slightly increased overall risk [12]. Two studies stated more clearly elevated overall risks for breast cancer with OR = 1.31 (95% CI 1.03–1.65) [19] and RR = 2.6 (95% CI 1.4–4.7) [14] respectively.

As there is a lot of research on progestin-only methods other than DMPA, a comparison to these contraceptives thus seemed appropriate. Several studies examined the relationship between progestin-only contraceptives and breast cancer risk, but the findings were mixed. Some found an increased risk of breast cancer in women who used progestin-only contraceptives, while others found no significant association.

Regarding the progestin-only pill, there was evidence to suggest that they may slightly increase the risk of breast cancer, at least with current or recent use. The overall risk, however, was still considered relatively low [22–24].

The same applies to hormone replacement therapy (HRT) with medroxyprogesterone acetate (MPA). The association between HRT and breast cancer has been a topic of extensive research and debate. Recent research has provided a more nuanced understanding of this relationship. Several studies indicated that the long-term use of combined estrogen-progestin therapy (including MPA) seemed to be associated with a slight increase in the risk of breast cancer, while the use of estrogen alone significantly reduced the breast cancer incidence. Nonetheless, the absolute breast cancer risk remained small [25–27].

A recent systematic review showed that the breast cancer risk seemed to be slightly elevated in users of the levonorgestrel-releasing intrauterine system (LNG-IUS). This was especially the case in postmenopausal women and with longer durations of use [28].

Lastly, there is limited evidence on progestin-only implantable devices. Although the numbers of exposed women were small, implants did not seem to increase the breast cancer incidence significantly [18].

As stated before, most of the studies on DMPA and breast cancer included in this review could not find an increased risk for exposed women. However, there was a trend towards a slightly elevated incidence in younger women who received DMPA currently or more recently.

An explanation for this finding could be that women younger than 35 years were more likely to have used DMPA recently. A further source of bias could be due to enhanced detection of breast tumors in women using DMPA, as they may be advised to pay closer attention to their breast health and healthcare providers may encourage them to perform regular breast self-examinations more frequently. In this way, DMPA users would be (over)diagnosed earlier and show more cases in the younger age groups, as well as in current and recent users.

The results of a study investigating potential bias in case–control studies on OCs and breast cancer could at least to some extent also be applicable for the selected studies in this review [29]. The question was asked whether hospital controls are a suitable comparison group: Some studies [12, 17, 19] included in this systematic review used women hospitalised for other reasons than breast cancer as controls, which might not have represented the actual population accurately. Controls selected from nationwide surveys or electoral rolls might be a better representation, this was the case for two studies [14, 16]. Telephone interviews could also be more suitable than in-person questionnaires, as the interviewer is blind to whether the woman is a case or a control patient, at least initially.

Two studies showed obvious methodological flaws with a short average exposure, small numbers of cases and a low statistical power. Data from fertility or family planning clinics could also be susceptible to bias [13, 21].

Other systematic reviews on the influence of DMPA and progestins on breast cancer largely agree with the conclusions drawn in the present review. Although the authors compared fewer papers, they also found no overall risk increase for DMPA users [30, 31].

To the best of our knowledge, this study is one of the first to comprehensively review the existing literature on DMPA and breast cancer, including recent studies on this topic. However, a further meta-analysis would also have been useful to better evaluate the methodological and statistical approach of the analysed literature.

Finally, the benefits of DMPA such as the high effectiveness and long-lasting but reversible contraception outweigh the potentially slightly increased risk of breast cancer in younger women or current users. Even so, women should be informed – as with all contraceptive methods – about the possible negative effects of DMPA use.

## Conclusion

In summary, there was little evidence that DMPA exposure may increase the overall risk for breast cancer. It should be noted, however, that there were indications that the incidence of breast cancer was possibly increased in current and recent users, especially in women younger than 35 years. Longer duration of use did not appear to elevate the overall risk. Nevertheless, these conclusions can not be assessed conclusively yet, as the data were still too sparse, and the results were inconsistent with one another. Further studies will be necessary to confirm these findings and weigh up the individual risks and benefits of this contraceptive method.

## Data Availability

As this is a systematic review there are no original data that could be provided.
